# *Bordetella* Adenylate Cyclase Toxin Differentially Modulates Toll-Like Receptor-Stimulated Activation, Migration and T Cell Stimulatory Capacity of Dendritic Cells

**DOI:** 10.1371/journal.pone.0104064

**Published:** 2014-08-01

**Authors:** Irena Adkins, Jana Kamanova, Aneta Kocourkova, Martina Svedova, Jakub Tomala, Hana Janova, Jiri Masin, Barbora Chladkova, Ladislav Bumba, Marek Kovar, Padraig J. Ross, Ludmila Tuckova, Radek Spisek, Kingston H. G. Mills, Peter Sebo

**Affiliations:** 1 Laboratory of Molecular Biology of Bacterial Pathogens, Institute of Microbiology of the Academy of Sciences of the Czech Republic, Prague, Czech Republic; 2 Laboratory of Tumor Immunology, Institute of Microbiology of the Academy of Sciences of the Czech Republic, Prague, Czech Republic; 3 Laboratory of Specific Cellular Immunity, Institute of Microbiology of the Academy of Sciences of the Czech Republic, Prague, Czech Republic; 4 Institute of Immunology, Charles University, 2^nd^ Faculty of Medicine and University Hospital Motol, Prague, Czech Republic; 5 Immune Regulation Research Group, School of Biochemistry and Immunology, Trinity College, Dublin, Ireland; Universidad Nacional de La Plata., Argentina

## Abstract

Adenylate cyclase toxin (CyaA) is a key virulence factor of the whooping cough agent *Bordetella pertussis*. The toxin targets CD11b-expressing phagocytes and delivers into their cytosol an adenylyl cyclase (AC) enzyme that subverts cellular signaling by increasing cAMP levels. In the present study, we analyzed the modulatory effects of CyaA on adhesive, migratory and antigen presenting properties of Toll-like receptor (TLR)-activated murine and human dendritic cells (DCs). cAMP signaling of CyaA enhanced TLR-induced dissolution of cell adhesive contacts and migration of DCs towards the lymph node-homing chemokines CCL19 and CCL21 *in vitro*. Moreover, we examined in detail the capacity of toxin-treated DCs to induce CD4^+^ and CD8^+^ T cell responses. Exposure to CyaA decreased the capacity of LPS-stimulated DCs to present soluble protein antigen to CD4^+^ T cells independently of modulation of co-stimulatory molecules and cytokine production, and enhanced their capacity to promote CD4^+^CD25^+^Foxp3^+^ T regulatory cells *in vitro*. In addition, CyaA decreased the capacity of LPS-stimulated DCs to induce CD8^+^ T cell proliferation and limited the induction of IFN-γ producing CD8^+^ T cells while enhancing IL-10 and IL-17-production. These results indicate that through activation of cAMP signaling, the CyaA may be mobilizing DCs impaired in T cell stimulatory capacity and arrival of such DCs into draining lymph nodes may than contribute to delay and subversion of host immune responses during *B. pertussis* infection.

## Introduction

Despite extensive vaccination programs, pertussis called also whooping cough, remains the least controlled vaccine-preventable infectious disease and represents a significant health burden world-wide, accounting for as many as 300 000 deaths per year [1]. The currently observed significant upsurge of pertussis incidence in the most developed countries then raises substantial prospective concern about evolution of whooping cough epidemiology [2]. This highly contagious disease is caused by the Gram-negative coccobacilli *Bordetella pertussis* and *B. parapertussis* that adhere to ciliated epithelial cells of human nasopharynx and trachea. The concerted action of virulence factors, such as adhesins and toxins, then enables bacteria to resist the clearance by the mucociliary escalator and host immune system [1], [3].

The adenylate cyclase toxin (CyaA) is a key virulence factor of *B. pertussis* that subverts host defense [4]. It has been shown that CyaA-deficient bacteria are unable to cause lethal infection and are cleared rapidly from the lungs in a mouse challenge model [5]. The 1706 aa long protein carries an N-terminal adenylate cyclase (AC) domain (∼400 residues) that penetrates into host phagocyte cytosol, eliciting Ca^2+^ influx [6]. Upon binding of intracellular calmodulin, the AC catalyzes conversion of ATP to cAMP, a key second messenger that subverts bactericidal functions of phagocytes. The C-terminal RTX hemolysin (Hly) moiety (∼1306 residues) then mediates CyaA toxin binding to myeloid phagocytic cells via the α_M_β_2_ integrin, known also as CD11b/CD18, complement receptor 3 (CR3), or Mac-1) [7]. The Hly moiety permeablizes target cell membranes by forming cation-selective toxin pores, thus perturbing ion homeostasis [4], [8]. CyaA-induced efflux of K^+^ ions from the host cell was, indeed, shown to activate the NALP3 inflammasome and promote IL-1β release from LPS-primed dendritic cells (DCs) [9].

CyaA-induced cAMP signaling quickly incapacitates anti-bacterial functions of macrophages and neutrophils by inhibiting superoxide production, chemotaxis and phagocytosis [10], [11] and promotes subsequent apoptosis [12] or necrosis [13]. We observed that by causing transient decrease of RhoA activity, the CyaA provokes subversive membrane ruffling and actin cytoskeleton rearrangements in macrophages, which is accompanied by an immediate shut-down of macropinocytosis [14].

Furthermore, cAMP signaling of CyaA was shown to selectively modulate Toll-like receptor (TLR)-induced activation and maturation of DCs, enhancing IL-10 and inhibiting IL-12p70 production, respectively, and promoting expansion of IL-10-secreting regulatory T cells (Tr1) [15]–[19]. CyaA activity was further reported to modulate Th1/Th17 polarization induced by *B. pertussis*-treated DCs towards enhanced Th17 and limited Th1 expansion [20]. Th17 and Th1 cells were shown to be involved in clearance of *B. pertussis* from the respiratory tract in mice immunized with a whole cell pertussis vaccine (Pw) [21]. Moreover, IL-1β-induced Th17 cells have been shown to play a critical role in clearance of a primary infection with *B. pertussis* [9]. Although it has been suggested that CD8^+^ T cells are dispensable for protective immunity to this bacterium [22], [23], it has been recently shown that CD8^+^ T cells participate in the immune response to acute *B. pertussis* infection [24] and pertussis-specific CD8^+^ memory T cells are induced by vaccination in humans [24], [25].

Here, we extended the studies on immunomodulatory action of CyaA on TLR-activated mouse bone-marrow derived DCs (BMDCs) and human monocyte-derived DCs (MDDCs) using a close to physiologically low toxin concentration [26]. We show that CyaA accelerates LPS-induced cell detachment and migration towards the lymph node-homing cytokines CCL19 and CCL21 *in vitro*. Such DCs exhibited a decreased capability to stimulate proliferation of antigen-specific CD4^+^ and CD8^+^ T cells *in vitro* and *in vivo*, which was independent of their capacity to engulf and degrade protein antigens. Moreover, CyaA treatment of DCs decreased their ability to induce IFN-γ-secreting CD8^+^ T cells, but promoted antigen-specific IL-10 and IL-17-producing CD8^+^ T cells, and enhanced the relative numbers of CD4^+^CD25^+^Foxp3^+^ T regulatory cells.

## Materials and Methods

### Mice

6–8 weeks old C57BL/6 (Ly5.2) mice were purchased from Charles River Laboratories, Germany. C57BL/6 (Ly5.1) and OT-I mice were a generous gift of Marek Kovar, Institute of Microbiology of ASCR, v.v.i. OT-II mice were a generous gift of Thomas Jacobs, Bernhard-Nocht-Institute for Tropical Medicine, Hamburg, Germany and Pavel Otahal, Institute of Molecular Genetics of ASCR. All of the experimental procedures were approved by the Animal Welfare Committee at the Institute of Microbiology of ASCR in accordance with institutional and state guidelines on animal welfare and every effort was made to minimize suffering.

### Generation of mouse and human dendritic cells

Bone marrow-derived DCs (BMDCs) were generated according to Lutz et al. (1999) [27]. Briefly, bone marrow cells were flushed from femurs and tibias of mice, and cultured at 2×10^6^/ml in 100-mm dishes in 10 ml of RPMI 1640 medium supplemented with 10% (v/v) heat-inactivated fetal calf serum (FCS) (Life Technologies), 0.1 mg/ml streptomycin, 100 U/ml penicillin and 0.25 µg/ml amphotericin (Sigma-Aldrich), 50 µM 2-mercaptoethanol, 1% non-essential amino acids (Biochrom), 1 mM sodium pyruvate, 2 mM glutamine and 200 U/ml granulocyte-macrophage colony-stimulating factor (GM-CSF; PeproTech). Fresh medium was added on day 3 or changed on day 6. Loosely attached cells were used for experiments at days 6–8. 70–80% of cultured cells at day 6–8 expressed CD11c and 90% CD11b. Before performing experiments, the BMDC's phenotype was checked for expression levels of CD11c+, CD11b+, I-A/I-E+, Gr-1, F4/80 and B220 low/negative (Fig. S1A).

Immature monocyte-derived DCs (MDDCs) were generated as previously described [28]. Briefly, human PBMC were isolated from buffy coats of healthy donors (provided by the Department of Blood Transfusion at Thomayer's Hospital, Prague, Czech Republic or Institute of Hematology and Blood Transfusion, Prague, Czech Republic) by Ficoll-Paque plus gradient centrifugation (GE Healthcare). PBMC at the concentration of 3×10^6^ cells/ml were incubated in 75 cm^2^ plastic culture flasks (Nunc). After 2 h, the non-adherent fraction of cells was washed away and isolated adherent monocytes were cultured in the presence of human GM-CSF (500 U/ml; Gentaur) and recombinant human IL-4 (20 ng/ml; PeproTech) in RPMI 1640 (BioWhittaker, Lonza), supplemented with L-glutamine (2 mM, Sigma), penicillin/streptomycin (100 U penicillin/ml, 100 µg streptomycin/ml), and 10% FCS (BioWhittaker, Lonza) at 37°C. Immature DCs were harvested on day 5 of culture. The phenotype of MDDCs had been verified before starting experiments (CD11c+, HLA-DR+, CD1a+, CD14 low/negative) (Fig. S1B). To avoid uncontrollable chelation of calcium ions by the phosphate ions contained in RPMI 1640 medium, DC stimulations were performed in DMEM medium as calcium is required for CyaA activity.

### Purification of CyaA and determination of intracellular cAMP and cell viability

Wild type CyaA and CyaA-AC^−^ (CyaA mutant devoid of adenylate cyclase activity) were purified as described from *E. coli* [29]. The endotoxin content in samples was determined by the Limulus amebocyte lysate assay (QCL-1000; Cambrex) and was below 200 EU/mg of purified protein.

For determination of intracellular cAMP, BMDCs or MDDCs (2×10^5^/sample) were seeded in 96-well plate in RPMI media containing 10% FCS and allowed to attach for 2 h at 37°C. Subsequently, RPMI media with 10% FCS was replaced by 150 µl DMEM without FCS and the CyaA at 10 ng/ml was added for 30 min. The reaction was stopped by addition of 0.2% Tween 20 in 50 mM HCl, and the samples were boiled for 15 min at 100°C to denature cellular proteins (cAMP is resistant to acid and heat). The samples were neutralized by addition of 150 mM unbuffered imidazole, and the concentration of cAMP was determined by a competition ELISA performed as previously described [30].

To determine cell viability, BMDCs (3×10^5^/sample) or MDDCs (1×10^6^/sample) were left untreated or incubated for 18 h or 24 h, respectively, with 10 ng/ml CyaA or CyaA-AC^−^ and LPS (1 µg/ml for MDDCs or 100 ng/ml for BMDCs, *E. coli* 0111:B4, Sigma-Aldrich) and subsequently stained with Annexin-V-FITC (BD Pharmingen) and 0.5 µg/ml Hoechst 33258 (Invitrogen). The necrotic and/or apoptotic cells were detected by flow cytometry using FACS Aria (MDDCs) or LSR II instruments (BD Biosciences) (BMDCs) and analyzed by flow cytometry software (FlowJo Version 8.8.7, Tree Star, Inc.). In some experiments higher concentrations of 100 ng/ml or 300 ng/ml of CyaA and CyaA-AC^−^ were used.

### Determination of cell adhesion and spreading

To determine the adhesion and spreading of MDDCs impedance measurements using xCelligence system in E-plates (Roche) were performed. The increase in cell spreading and adhesiveness leads to increase in impedance, as cells attached on the electrode sensor surfaces act as insulators [31]. E-plates were coated with fibronectin in PBS for 1 h at room temperature, washed with PBS and background was determined in 90 µl of DMEM medium supplemented with 10% FCS using Real-Time Cell Analyzer (RTCA) station. Subsequently, MDDC suspension in DMEM medium (1×10^5^/well), and LPS (1 µg/ml) alone or in combination with 10 ng/ml CyaA or CyaA-AC^−^ was added. Cells in E-plates were placed in the RTCA station for 24 h cultivation at 37°C in a 5% CO_2_ atmosphere. Impedance was measured every 5 min for an initial 5 h period of cultivation, and every 15 min for the remaining period of 19 h. Impedance was represented by the cell index (CI) values (R_i_-R_0_) [Ohm]/15 [Ohm]; R_0_: background resistance, R_i_: individual time point resistance).

### DC migration

DCs were left untreated, or treated with LPS (1 µg/ml for MDDCs or 100 ng/ml for BMDCs) alone or in combination with 10 ng/ml CyaA or CyaA-AC^−^ at cell density 1×10^6^/ml in DMEM medium supplemented with 10% FCS for 24 h. Subsequently, cells were washed and their migration was measured in 96-well Transwell cell culture chambers with 5 µm pore size polycarbonate filters (Corning Costar). The lower chambers of the Transwell plates were filled with 235 µl of RPMI 1640 medium with or without CCL19 or CCL21 (200 ng/ml; Peprotech), and in total, 1×10^5^ DCs diluted in 75 µl of RPMI medium were deposited in the upper chamber. After 14 h (MDDCs) or 4 h (BMDCs) of incubation at 37°C in a 5% CO_2_ atmosphere, the Transwell inserts were removed. DCs in the lower chamber were transferred into a new 96-well plate, stained with Hoechst 33258 and live cells were counted by flow cytometry. CCR7 expression was determined by using CCR7-PE antibody (eBiosciences).

### Measurement of antigen uptake, processing and degradation

To measure antigen (Ag) uptake, DCs (3×10^5^/sample) were left untreated, or pretreated with LPS (100 ng/ml) and 10 ng/ml of CyaA or CyaA-AC^−^ for 30 min. Subsequently, OVA-Alexa647 or transferrin-Alexa647 (both 5 µg/ml, Invitrogen) or Lucifer yellow (500 µg/ml, Invitrogen) were added for 30 min at 37°C. The Ag uptake was assessed by flow cytometry.

For MHC class I-restricted processing DCs (2×10^6^/sample) were left untreated, or incubated with 10 ng/ml of CyaA or CyaA-AC^−^ ng/ml or lactacystin (10 µM, Sigma-Aldrich) and LPS (100 ng/ml) for 30 min. Protein concentration in lysate was determined by MicroBCA™ Protein Assay kit (Pierce). 50 µg of proteins in 20 mM Tris-HCl, pH 7.4 was mixed with 100 µM Z-Leu-Leu-Glu-AMC, Suc-Leu-Leu-Val-Tyr-AMC or Boc-Leu-Arg-Arg-AMC fluorogenic substrates (BIOMOL) and incubated for 90 min at 37°C. The fluorescence of liberated 7-amino-4-methylcoumarin was measured using a microplate reader (380_ex_/460_em_, Safire^2^, Schoeller Instruments).

For MHC class II-restricted Ag processing DCs (1×10^6^/sample) were left untreated, or incubated with 10 ng/ml of CyaA or CyaA-AC^−^ or chloroquine (100 µM, Sigma-Aldrich) and LPS (100 ng/ml) for 30 min. DCs were subsequently loaded with a mixture of OVA-Alexa647 and OVA labeled with BODIPY FL dye (OVA-DQ, both 5 µg/ml, Invitrogen) for 30 min at 37°C and analyzed by flow cytometry [32].

### T cell expansion *in vitro* and *in vivo*

For T cell proliferation *in vitro* naïve OVA-specific CD8^+^ or CD4^+^ T cells were isolated from lymph nodes and spleen of OT-I or OT-II transgenic mice, respectively, by a magnetic cell separation using CD8^+^ and CD4^+^ T cell Isolation kits (Miltenyi) and labeled with 3 µM CFSE (Invitrogen) for 10 min, at 37°C. 2×10^5^ T cells were added to washed 5×10^4^ DCs which had been left untreated, or pretreated with LPS (100 ng/ml) and 10 ng/ml of CyaA or CyaA-AC^−^ and OVA protein at 2.5 µg/ml (OT-II) or at 5 µg/ml (OT-I) for 4 h at 37°C. In some experiments 5 µg/ml OVA_323–339_ peptide (ISQAVHAAHAEINEAGR, GeneScript) or 1 ng/ml OVA_257–264_ peptide (SIINFEKL) were added instead of the OVA protein for 4 h at 37°C. After 72 h of incubation T cells were stained by CD8-PE (53–6.7, eBioscience) or CD4-PE (RM4-4, eBioscience) antibodies and their proliferation was determined as a CFSE dilution by flow cytometry.

For T cell expansion *in vivo*, purified CFSE-labeled T cells (Ly5.2) were injected intravenously (i.v.) into C57BL/6 recipients (Ly5.1) at 1.5×10^6^ cells per mouse. 24 h later mice were injected i.v. with 1.5×10^6^ DCs that had been pretreated with toxin, LPS and OVA peptides as described above. Control mice were injected intraperitoneally (i.p.) with PBS or OVA peptide+poly I:C (75 µg). Three days later, spleen cells were harvested, fixed by 4% paraformaldehyde and stained with CD8-A700 or CD4-PerCP and Ly5.2-APC antibody (BD Pharmingen). The expansion of adoptively transferred T cells was determined by flow cytometry.

MDDCs, derived from monocytes of HLA-A2 positive healthy donors, were left untreated, or stimulated with LPS (1 µg/ml) alone or in combination with 10 ng/ml of CyaA or CyaA-AC^−^ at cell density 10^6^/ml in DMEM medium supplemented with 10% FCS for 24 h. Subsequently, DCs were pulsed with the HLA-A2 restricted influenza matrix peptide (aa 58–66, GILGFVFTL) for 2 h, washed and added to autologous lymfocytes at T cell: DC ratio of 10 : 1 for 7 days. IL-2 (50 U/ml) was added on day 3. On day 7, lymphocytes were re-stimulated with fresh peptide-loaded DCs for 1 h and analyzed for IFN-γ production by intracellular flow cytometry staining, as follows: Brefeldin A (e-Bioscience, San Diego, CA, USA) was added to block the release of IFN-γ After 3 h cells were stained with CD3-Alexa700 and CD8-PE-Dy590 antibodies (Exbio) at 4°C for 20 minutes. Subsequently, cells were fixed and permeabilized using Fixation and Permeabilization Buffers (e-Bioscience), respectively, and stained with IFN-γ-FITC antibody (e-Bioscience). Samples were analyzed using flow cytometry.

### T cell responses and expansion of CD4^+^CD25^+^Foxp3^+^ T cells

BMDCs (5×10^4^/sample) were left untreated, or treated with 10 ng/ml of CyaA or CyaA-AC^−^ and/or LPS (100 ng/ml). Concomitantly OVA protein (2.5 µg/ml) was present for 4 h prior assays of MHC class II presentation or at 5 µg/ml prior assay of MHC class I presentation and incubated with T cells. Production of IL-17, IFN-γ and IL-10 by CD4^+^ and CD8^+^ T cells in supernatant was determined by ELISA after 72 h. Remaining CD4^+^ T cells were collected and stained with CD4-PerCP (BD Biosciences), CD25-APC and Foxp3-PE (eBioscience) using Fixation and Permeabilization buffers (eBioscience). The expression of Foxp3 in CD4^+^CD25^+^ T cells was determined by flow cytometry.

MDDCs were left untreated or incubated with LPS (1 µg/ml) alone or in combination with CyaA and CyaA-AC^−^ (10 ng/ml) for 24 h and used as stimulators of naïve allogeneic T cells, isolated by a negative selection using EasySep Human Naïve CD4^+^ T Cell Enrichment Kit (StemCell Technologies Inc.). T cells were used at T cell : DC ratio of 10 : 1 and IL-2 (50 U/ml) was added on day 3. After 7 days of co-culture, the frequency of CD4^+^ CD25^+^ Foxp3^+^ T regulatory cells was determined by flow cytometry. Cells were stained with CD8-PE-Dy590, CD25-PE (Exbio) and CD4-PE-Cy7 (eBioscience) antibodies at 4°C for 30 minutes, followed by cell fixation and permeabilization using Fixation and Permeabilization Buffers (e-Bioscience), respectively and staining with Foxp3-Alexa488 antibody (e-Bioscience). The gating strategy of T regulatory cells is shown in Fig. S4.

### Statistical analysis

Data were expressed as mean ± SEM. Statistical analysis was performed using software GraphPad (PRISM 6.0). The significance of the differences between groups was determined by using two-tailed Mann-Whitney U test. Differences were considered statistically significant if *p*<0.05 (*).

## Results

### Exposure to low concentration of CyaA does not induce cell death in TLR-activated DCs

**Figure 1:**
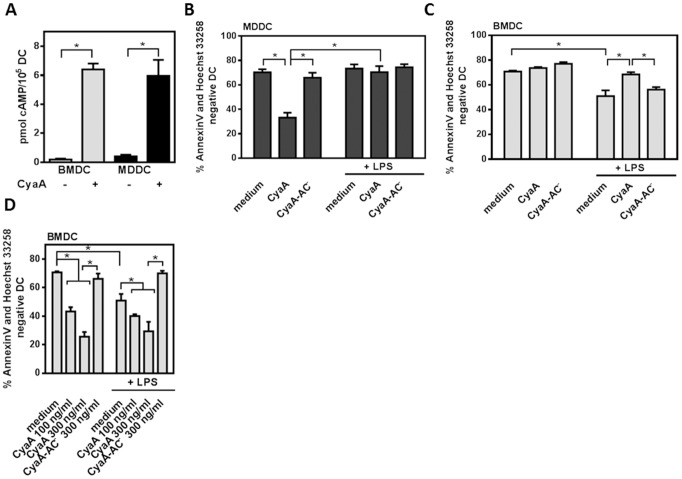
Exposure to low concentrations of CyaA does induce cell death in TLR-activated DCs. (A) BMDCs and MDDCs were left untreated or incubated with CyaA at 10 ng/ml for 30 min. The intracellular level of cAMP was determined by ELISA. (B, C) DCs were left untreated (medium), or incubated with LPS (1 µg/ml MDDCs or 100 ng/ml BMDCs), CyaA or CyaA-AC^−^ at 10 ng/ml alone or in their combination for 18 h (BMDCs) or 24 h (MDDCs) (D) BMDCs were incubated with LPS and CyaA or CyaA-AC^−^ at 100 ng/ml or 300 ng/ml alone or in their combination for 18 h and stained with Annexin V-FITC and Hoechst 33258. Values represent the means ± SEM of n = 3–5 or 5–6 donors, respectively (* *p*<0.05).

CyaA used at high concentrations was shown to induce cell death [12], [13]. Therefore, prior to analyzing the effect of CyaA on immunostimulatory activities of TLR-stimulated DCs, we first examined the impact of exposure to low CyaA concentration (10 ng/ml) on viability of murine BMDCs and human MDDCs. As shown in Fig. 1A, upon 30 min of incubation with 10 ng/ml of CyaA, the BMDCs and MDDCs accumulated similarly high levels of intracellular cAMP. Fig. 1B then shows that the treatment of MDDCs with 10 ng/ml of CyaA in the absence of LPS led to a significant cell death in 24 hours. However, co-treatment with LPS rescued MDDCs from CyaA-induced cell death. On the contrary, mouse BMDCs were more resistant to CyaA-induced cell death in the absence of LPS, as CyaA at 10 ng/ml did not reduce BMDC's viability (Fig. 1C). However, LPS treatment decreased the survival of BMDCs by 20% and the co-treatment of BMDCs with LPS and CyaA at 10 ng/ml rescued DCs from LPS-induced cell death. These effects were mediated by CyaA-induced cAMP signaling, since the enzymatically inactive CyaA-AC^−^ did not affect cell viability of untreated, or LPS-treated MDDCs and BMDCs, respectively. Fig. 1D further shows that CyaA at the higher concentrations of 100 ng/ml and 300 ng/ml induced cell death in BMDCs independently of LPS-signaling. The cytotoxic effects of LPS or CyaA varied depending on the origin of DCs. Interestingly, the co-incubation with both CyaA (10 ng/ml) and LPS lead to pro-survival signaling in both types of DCs, MDDCs and BMDCs.

### CyaA accelerates TLR-mediated dissolution of cell adhesive contacts and migration of DCs

**Figure 2:**
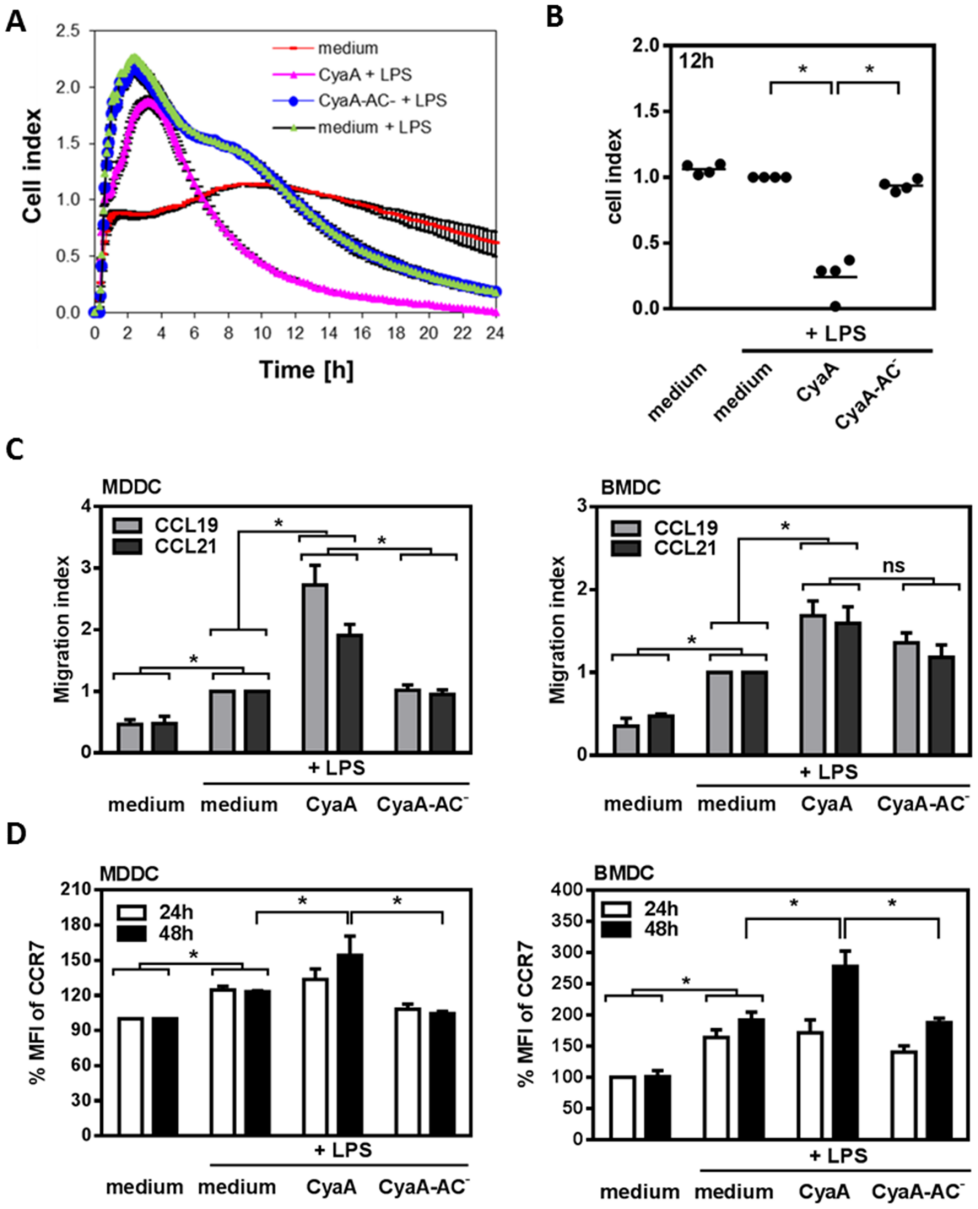
CyaA accelerates cell detachment and migration of TLR-activated DCs. (A) Impedance measurements using the real-time cell electronic sensing system xCelligence were used to determine MDDC adhesion and spreading. MDDCs were seeded on fibronectin-coated sensors and were left untreated (medium), or treated with LPS (1 µg/ml) alone, or in combination with CyaA or CyaA-AC^−^ at 10 ng/ml for 24 h. The representative experiment is shown (A) as well as quantitative analysis of 4 donors at time point of 12 h (B) where cell index (CI) of LPS-treated DCs at 12 h was normalized to 1.0. (C) Migration of DCs treated with toxins and LPS (for 24 h) towards CCL19 or CCL21 (both 200 ng/ml) in transwell plates was determined by flow cytometry after additional 14 h (MDDCs) or 4 h (BMDCs) of incubation at 37°C. Values represent the means ± SEM of n = 4 or 5 donors, respectively (* *p*<0.05) where the number of transmigrated LPS-treated DCs (medium) was set to 1. (D) CCR7 expression on DCs was determined by flow cytometry after 24 h and 48 h. Values represent the means ± SEM of n = 3–5 or 5 donors, respectively (* *p*<0.05).

We have previously observed that as low doses of CyaA as 10 ng/ml induce massive actin cytoskeleton rearrangements and ruffling of mouse macrophages [14]. Therefore we analyzed the effects of exposure to low doses of CyaA (10 ng/ml) on the immune functions of DCs. Modulation of human or mouse DC maturation and IL-12p70 and IL-10 cytokine production by CyaA has been extensively analyzed *in vitro* using either the *E. coli* LPS, or in the context of DC infection by *B. pertussis* strains [15]–[19]. CyaA at higher concentrations was shown to inhibit LPS-stimulated CD40 expression and IL-12p70 production and enhance IL-10 secretion from both mouse and human DCs [15]–[19]. As shown in Fig. S2, treatment with CyaA (10 ng/ml) reproduced the previously reported impact of CyaA action on DC phenotype and cytokine production in the presence of LPS [15]–[19]. We extended, therefore, our analysis to CyaA effects on activation, adhesive and migratory properties of DCs after TLR-stimulation. We first assessed DC adhesion and spreading by impedance measurements using the xCelligence system [31]. MDDCs were left untreated, or treated with LPS alone, or in combination with CyaA or CyaA-AC^−^ at 10 ng/ml for 24 h. As shown in Fig. 2A, the impedance measurements revealed that LPS stimulation of MDDCs resulted in rapid increase of cell adhesion and spreading, as detected by a rise in the cell index (CI). This was followed by drop of the CI value below that of control non-treated cells (medium) within 24 h, due to progressing maturation and cell rounding up. Importantly, addition of CyaA to LPS-activated MDDCs resulted in a faster decrease of cell adhesion and spreading, as compared to LPS stimulated cells and cells treated with LPS and non-enzymatic CyaA-AC^−^ toxoid (Fig. 2A). Fig. 2B then depicts the CI values of the samples at 12 h of the impedance measurements. These results suggest that through elevation of cAMP the CyaA action accelerates TLR-induced decrease of cell adhesion that accompanies DC maturation.

Reorganization of the cytoskeleton and dissolution of cell adhesive contacts during DC maturation is important for the capacity of DCs to migrate into the lymph nodes. Therefore, we next examined whether the increased dissolution of cell adhesion mediated by CyaA also leads to an increased migration of TLR-stimulated DCs to CCL19 or CCL21 chemokines. BMDCs or MDDCs were exposed for 24 h to LPS alone or in combination with 10 ng/ml of CyaA or CyaA-AC^−^ and the cells were allowed to migrate across a transwell membrane into medium with chemokines for additional 14 h (MDDCs) or 4 h (BMDCs), respectively. As shown in Fig. 2C, CyaA increased chemotactic responsiveness of LPS-stimulated DCs to CCL19 or CCL21 chemokines compared to cells treated with LPS and CyaA-AC^−^ toxoid. However, after 24 h of treatment with CyaA and LPS, the DCs did not express significantly higher amounts of the CCL19/21 chemokine receptor CCR7 than DCs treated with LPS alone (Fig. 2D). As shown in Fig. 2D, a significantly increased expression of CCR7 on DCs treated with LPS and CyaA toxin was observed only after 48 h of incubation.

### CyaA decreases the capacity of TLR-stimulated DCs to present soluble antigens to CD4^+^ T cells

**Figure 3:**
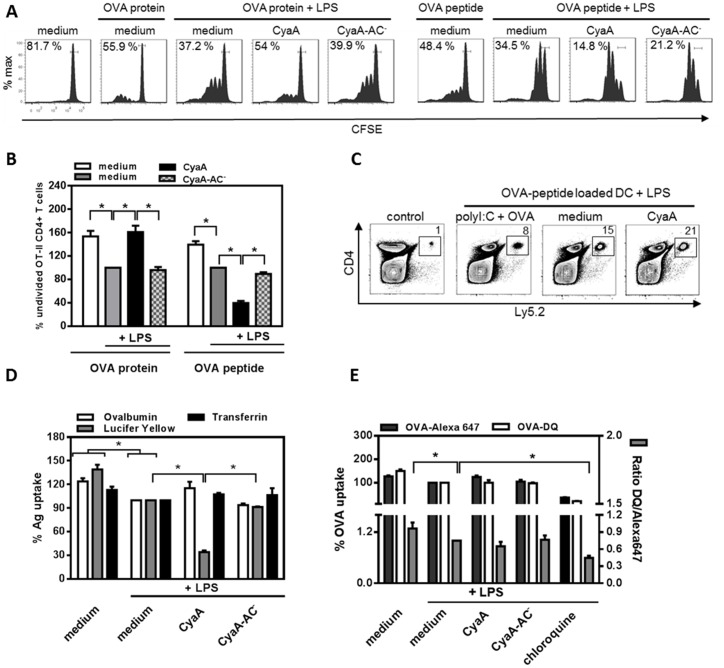
CyaA decreases the capacity of TLR-stimulated DCs to present soluble antigen to CD4^+^ T cells. BMDCs were left untreated, incubated with LPS (100 ng/ml) alone or in combination with CyaA or CyaA-AC^−^ at 10 ng/ml in the presence of OVA protein at 2.5 µg/ml or OVA_323–339_ peptide (5 µg/ml) for 4 h prior to washing and co-cultivation with naïve CFSE-labeled OT-II CD4^+^ T cells. T cell proliferation was determined by flow cytometry after 72 h as a dilution of CFSE. (A) Histograms are representative of n = 4. (B) Quantitative analysis of A where the percentage of undivided LPS-treated cells (medium) was set to 100% (* *p*<0.05). (C) Expansion of adoptively transferred CFSE-labeled CD4^+^ T cells *in vivo* was determined after 72 h by flow cytometry as a fold of expansion of 2×10^6^ counted spleen cells where 1 represents the non-stimulated adoptively transferred CD4^+^ T cells (control). Dot plots are representative of n = 3. (D, E) CyaA inhibits macropinocytosis but not receptor-mediated endocytosis and antigen (Ag) degradation in LPS-treated DCs. DCs were left untreated, incubated with LPS alone or in combinantion with 10 ng/ml of toxins or chloroquine (100 µM) for 30 min. (D) Lucifer Yellow (500 µg/ml), transferrin-Alexa647 or OVA-Alexa647 (both 5 µg/ml) were subsequently added for 30 min. The Ag uptake in living CD11c^+^ cells was determined by flow cytometry. (E) A mixture of OVA-Alexa647 (5 µg/ml, marker for Ag uptake) and OVA-DQ (5 µg/ml, marker for Ag uptake and degradation) were added for 30 min. The processed OVA-DQ was determined from gated CD11c^+^OVA-DQ^+^OVA-Alexa647^+^ DCs and calculated as a ratio of MFI OVA-DQ/OVA-Alexa647. Values represent means ± SEM of n = 5 where Ags taken up by LPS-treated DC (medium) was set to 100% of MFI (ratio 1) (* *p*<0.05).

CyaA differentially modulates LPS-stimulated DC maturation and cytokine production [16], [18], [19], which may affect their capacity to stimulate T cells. Therefore, we further investigated the capacity of CyaA-treated DCs to stimulate proliferation of CD4^+^ T cells. BMDCs were left untreated, or treated with 10 ng/ml of CyaA or CyaA-AC^−^ in the presence of ovalbumin (OVA) and LPS for 4 h. Toxin was removed by cell washing and naïve OVA-specific OT-II CD4^+^ T cells were then added. As shown in Fig. 3A and 3B, pretreatment of DCs with CyaA decreased their capacity to stimulate T cell proliferation, as higher percentage of undivided OT-II CD4^+^ T cells (54%) was detected by a CFSE dilution assay at 72 hours after CD4^+^ T cell incubation with CyaA-treated and OVA-loaded DCs, than after incubation with DCs treated with LPS only (37.2%), or with the CyaA-AC^−^ plus LPS-treated DCs (39.9%), respectively.

To investigate whether absence of co-stimulation or the CyaA-induced production of inhibitory soluble factors like IL-10 (Fig. S2) accounted for the reduced capacity of CyaA-pretreated DCs to stimulate CD4^+^ T cells, we used CyaA-pretreated DCs loaded with specific OVA peptide, which in contrast to a protein antigen does not require processing for T cell presentation. As further shown in Fig. 3A and 3B, the CyaA-pretreated DCs loaded with the OVA peptide stimulated even higher CD4^+^ T cell proliferation than control LPS- or CyaA-AC^−^-treated DCs. Similar data were obtained in an assay that assessed the expansion of adoptively transferred OT-II CD4^+^ T cells *in vivo* (Fig. 3C), where LPS-stimulated and CyaA-pretreated and OVA peptide-pulsed DCs induced higher proliferation of CD4^+^ T cells than control cells treated with LPS. This indicates that cAMP signaling of CyaA did not affect the overall capacity of LPS-treated DCs to stimulate CD4^+^ T cells when loaded with the OVA-derived peptide. However, the decrease in protein antigen presentation suggests that CyaA/cAMP may have affected antigen processing in LPS-stimulated DCs.

Using J774 macrophages we have previously shown that CyaA inhibited macropinocytosis in CD11b-expressing phagocytes [14] and this may have also impacted on presentation of OVA protein to T cells by DCs [33]. Therefore, we analyzed if CyaA action inhibits uptake of OVA. At the highest OVA concentration used in our study (5 µg/ml) the uptake of OVA was mediated solely by receptor-mediated endocytosis (Fig. S3). At higher concentrations, however, OVA could be internalized by both, macropinocytosis and receptor-mediated endocytosis [33]. We examined separately the effect of CyaA activity on macropinocytosis in DCs and on receptor-mediated endocytosis using Lucifer Yellow or Transferrin-Alexa647 uptake assays, respectively. BMDCs were left untreated, or exposed to 10 ng/ml of CyaA or CyaA-AC^−^ for 30 min in the presence of LPS, and then incubated with Ags for additional 30 min before being analyzed by flow cytometry. As shown in Fig. 3D, while CyaA action inhibited macropinocytosis of Lucifer yellow by ∼60%, it did not affect receptor-mediated endocytosis of transferrin in DCs compared to CyaA-AC^−^ and LPS-treated cells.

Since elevated cAMP levels were previously reported to reduce Ag degradation capacity of macrophages [34], we next examined if CyaA could inhibit Ag degradation in DCs. To analyze MHC class II-restricted processing, LPS and CyaA-treated DCs were incubated with a mixture of OVA-Alexa647, used as a marker for Ag uptake, and OVA labeled with BODIPY®FL dye (OVA-DQ), which served as a marker for Ag uptake and processing. Calculation of OVA-DQ degradation, was then determined as a ratio of % MFI OVA-DQ/% MFI OVA-Alexa647 by flow cytometry. As shown in Fig. 3E, however, only a slight and insignificant decrease in OVA-DQ degradation in DCs exposed to CyaA (0.78%±0.2) was observed, as compared to treatment with CyaA-AC^−^ toxoid (1.01%±0.2),), or to non-treated cells (1.0%±0.0). In contrast, endosomal processing was inhibited upon treatment with chloroquine (0.59%±0.05). These findings suggest that CyaA shuts down macropinocytosis but not the receptor-mediated endocytosis of OVA. As CyaA displayed no inhibitory effect on OVA uptake and degradation in LPS-stimulated DCs, the inhibition of OVA protein presentation to CD4^+^ T cells by CyaA-treated DCs is likely to involve other steps of antigen processing and presentation pathway within the DCs.

### CyaA commits DCs to expand CD4^+^CD25^+^Foxp3^+^ T regulatory cell population *in vitro*

**Figure 4:**
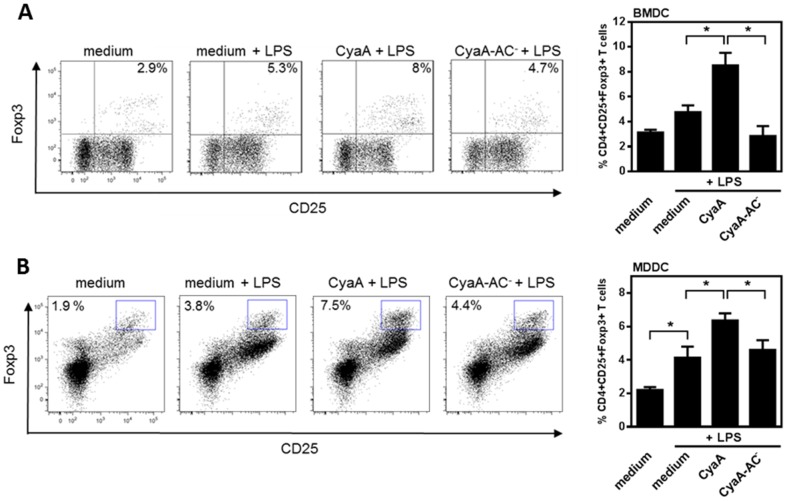
CyaA commits TLR-stimulated DCs to expand CD4^+^CD25^+^Foxp3^+^ T regulatory cells *in vitro*. (A) BMDCs were left untreated, incubated with LPS (100 ng/ml) alone or in combination with CyaA or CyaA-AC^−^ at 10 ng/ml in the presence of OVA at 2.5 µg/ml for 4 h prior to washing and co-cultivation with naïve CFSE-labeled OT-II CD4^+^ T cells. After 72 h the number of CD4^+^CD25^+^Foxp3^+^ T cells was determined by flow cytometry. Dot plots show one representative experiment and quantitative analysis represent means ± SEM of n = 4 (* *p*<0.05). (B) MDDCs were incubated with LPS (1 µg/ml) alone or in combination with CyaA or CyaA-AC^−^ at 10 ng/ml. After 24 h cells were used as stimulators of naïve allogeneic T cells at DC : T cell ratio of 1 : 10. The expansion of human CD4^+^CD25^+^Foxp3^+^ T regulatory cells was determined after 7 days. The representative experiment is shown and quantitative analysis represent means ± SEM of n = 5 (* *p*<0.05).

LPS-stimulated DCs treated with CyaA were shown to polarize IL-17-producing CD4^+^ T cells while reducing INF-γ production in T cells [18]. CyaA was also shown to induce IL-10 producing Tr1 cells [18]. However, it is not clear if LPS-stimulated DCs treated with CyaA expand a distinct subset of T regulatory cells, such as CD4^+^CD25^+^Foxp3^+^ T cells [35]. Therefore, BMDCs were left untreated, or incubated with LPS alone or in combination with CyaA or CyaA-AC^−^ at 10 ng/ml in the presence of OVA protein for 4 h before isolated naïve OVA-specific OT-II CD4^+^ T cells were added for another 72 h. The gating strategy used to detect both, human and mouse CD4^+^CD25^+^Foxp3^+^ T regulatory cells, is shown in Fig. S4. As documented in Fig. 4A by the results of flow cytometry analysis, CyaA significantly increased the capacity of LPS-stimulated BMDCs to expand OVA-specific CD4^+^CD25^+^Foxp3^+^ T cells as compared to CyaA-AC^−^-treated DCs.

Similarly, MDDCs were left untreated, or incubated with LPS alone, or in combination with CyaA or CyaA-AC^−^ (10 ng/ml) for 24 h before use for stimulation of naïve allogeneic T cells. Frequency of CD4^+^CD25^+^Foxp3^+^ T cells was measured by flow cytometry after 7 days. As shown in Fig. 4B, LPS and CyaA-treated MDDCs expanded the relative number of CD4^+^CD25^+^Foxp3^+^ T regulatory cells from co-cultivated CD4^+^ T cells. Collectively, these data show that CyaA-induced cAMP signaling skews the TLR-stimulated DCs towards the expansion of CD4^+^CD25^+^Foxp3^+^ T regulatory cells *in vitro*.

### CyaA reduces the capacity of TLR-stimulated DCs to induce CD8^+^ T cell proliferation

**Figure 5:**
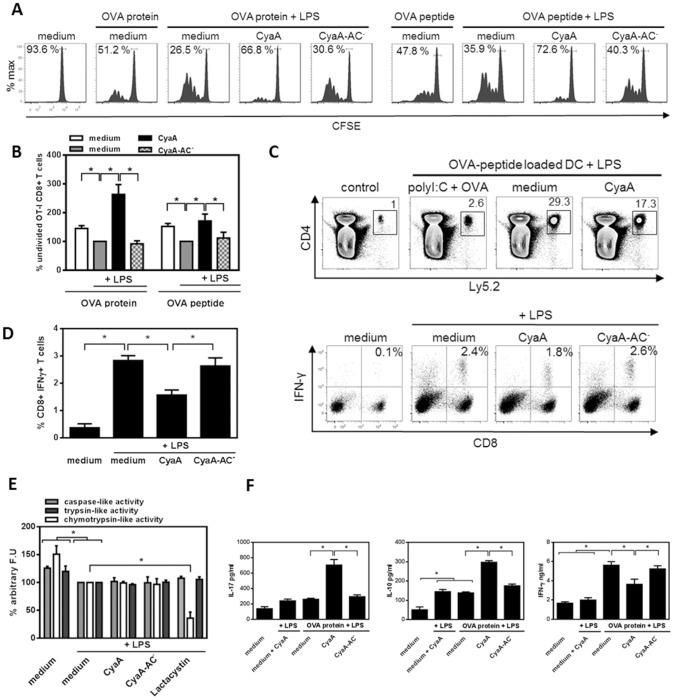
CyaA reduces the capacity of TLR-stimulated DCs to induce CD8^+^ T cell proliferation. BMDCs were left untreated, incubated with LPS (100 ng/ml) alone or in combination with 10 ng/ml of CyaA or CyaA-AC^−^ in the presence of OVA protein at 5 µg/ml or OVA_257–264_ peptide (1 ng/ml) for 4 h prior to co-cultivation with naïve CFSE-labeled OT-I CD8^+^ T cells. T cell proliferation was determined by flow cytometry after 72 h as a dilution of CFSE. (A) Histograms are representative of n = 4. (B) Quantitative analysis of A where the percentage of undivided LPS-treated cells (medium) was set to 100% (* *p*<0.05). (C) Expansion of adoptively transferred CFSE-labeled CD8^+^ T cells *in vivo* after 72 h was determined by flow cytometry as a fold of expansion of 2×10^6^ spleen cells counted where 1 represents the non-stimulated adoptively transferred CD8^+^ T cells (control). Dot plots are representative of n = 3 (D) MDDCs were incubated with LPS (1 µg/ml) alone or in combination with CyaA or CyaA-AC^−^ at 10 ng/ml for 24 h and then loaded with influenza matrix peptide. The induction of specific IFN-γ producting CD8^+^ T cells was determined after 7 days by flow cytometry. Dot plots show one representative experiment and quantitative analysis represent means ± SEM of n = 6 (* *p*<0.05). (E) BMDCs were left untreated, incubated with LPS (100 ng/ml) alone or in combination with CyaA or CyaA-AC^−^ at 10 ng/ml or inhibitor of proteasome lactacystin (10 µM) for 30 min. After cell lysis, 50 µg of cellular proteins was mixed with fluorogenic proteasomal peptide substrates (100 µM) and incubated 90 min at 37°C. Values represent means ± SEM of n = 4 where the amount of processed substrates by LPS-treated DCs (medium) was set to 100% (* *p*<0.05). (F) The production of IL-17, IL-10 and IFN-γ in BMDC-CD8^+^ T cell culture supernatant after 72 h was determined by ELISA. Mean values ± SEM are representative of n = 3 (* *p*<0.05).

To assess if the CyaA/cAMP-induced modulation of LPS-stimulated DCs impacts on activation of CD8^+^ T cell responses, BMDCs were left untreated, or pretreated with 10 ng/ml CyaA, or with CyaA-AC^−^, in the presence of OVA protein or peptide and LPS for 4 h. Residua ltoxin was removed by cell washing and naïve OT-I CD8^+^ T cells were added. As shown in Fig. 5A and 5B, treatment with CyaA interfered with the capacity of DCs to stimulate proliferation of CD8^+^ T cells. Higher percentage of undivided OT-I CD8^+^ T cells was detected by a CFSE dilution assay after incubation of CyaA-treated DCs for 72 h with OVA protein (66.8%), or with OVA peptide (72.6%), as compared to control LPS-treated DCs (26.5% or 35.9%, respectively) and the CyaA-AC^−^-treated DCs (30.6% or 40.3%),%), respectively. CyaA also decreased the capacity of LPS-stimulated DCs to induce proliferation of OT-I CD8^+^ T cells in response to OVA peptide after adoptive transfer *in vivo*, as compared to control LPS-treated and OVA peptide pulsed DCs (Fig. 5C).

Similarly, MDDCs, derived from monocytes of HLA-A2 positive healthy donors, were left untreated, or incubated with LPS alone or in combination with 10 ng/ml CyaA or CyaA-AC^−^ for 24 h. Subsequently, MDDCs were pulsed with the HLA-A2 restricted influenza matrix peptide (aa 58–66, GILGFVFTL) for 2 h, washed and incubated with autologous T lymphocytes for 7 days. IFN-γ-producing T cells were detected by flow cytometry after restimulation with such treated DCs. As shown in Fig. 5D, pretreatment with CyaA decreased the capacity of LPS-activated MDDCs to stimulate influenza specific CD8^+^ T cells compared to control LPS-treated or CyaA-AC^−^ and LPS-treated MDDCs, respectively.

To assess if CyaA impairs the MHC class I-restricted processing in DCs and this may account for the observed decrease in CD8^+^ T cell proliferation, lysates of BMDCs pretreated with CyaA or with CyaA-AC^−^ were incubated with fluorescent peptide substrates for 90 min and the proteolytic activities of the proteasome were determined as the intensity of resulting fluorescence. As shown in Fig. 5E, CyaA did not inhibit chymotrypsin-like, trypsin-like or caspase-like proteolytic activity of the proteasome, while this was inhibited by lactacystin, a known inhibitor of proteasomal proteolytic functions. These findings indicate that CyaA diminished the capacity of antigen-loaded DCs to stimulate CD8^+^ T cell proliferation *in vitro* as well as *in vivo*, which appeared to be independent of proteosomal Ag processing for MHC class I presentation.

In addition, the analysis of cytokine production in BMDC-CD8^+^ T cell culture supernatants by ELISA revealed that LPS and CyaA-pretreatment of DCs polarized the OVA-specific IL-17 and IL-10 production by CD8^+^ T cells, but suppressed production of IFN-γ (Fig. 5F). This goes well with the cytokine profiles induced by LPS and CyaA-pretreated DCs in CD4^+^ T cells (Fig. S5). These findings show that cAMP signaling of CyaA in DCs directs also CD8^+^ T cells to produce IL-17 and IL-10 and limits the antigen-specific production of IFN-γ in response to stimulation by LPS-activated DCs.

## Discussion

Through the elevation of cytosolic cAMP concentrations, the CyaA toxin exerts a remarkably complex set of effects on immune cell functions. This ranges from inhibition of phagocytosis [10], oxidative burst [11], or macropinocytic uptake [14], up to a direct cytotoxicity and apoptotic [12] or necrotic cell death [13]. Here we show that human monocyte-derived DCs (MDDCs), but not mouse bone marrow-derived DCs (BMDC), are highly sensitive to CyaA-mediated cytotoxicity already at as low concentration of CyaA as 10 ng/ml and that this cytotoxicity of CyaA could be counteracted by co-incubation with LPS. On the other hand, BMDCs were slightly more sensitive to LPS treatment and the addition of CyaA increased their survival rate *in vitro*. Interestingly, in the absence of LPS or other TLR ligands, CyaA was shown to induce phenotypic maturation of DCs and to enhance their capacity to stimulate T cells in mixed lymphocyte reaction [15]. Similarly, we have observed here that CyaA at 10 ng/ml also induced phenotypic maturation of BMDCs and MDDCs in the absence of TLR-stimulation (Fig. S6). However, this effect of LPS-free CyaA alone appears to be rather artificial, since in the context of *B. pertussis* infection a number of TLR ligands is shed by the infecting bacteria and the concerted action of other virulence factors would integrate into the final outcome as well. The main aim of this study was to analyze how low concentrations of CyaA shape the immune function of LPS-stimulated MDDCs and BMDCs. We confirmed that cAMP signaling induced by 10 ng/ml of *B. pertussis* CyaA toxin enhanced secretion of IL-10 and decreased production of IL-12p70 and CD40 expression by TLR-activated human and mouse DCs [16], [18], [19]. Moreover, as further shown here, it also enhanced TLR-induced cell detachment and chemotactic migration of DCs towards the chemokines CCL19/21. Furthermore, such subversively matured DCs exhibited a reduced capacity to stimulate antigen-specific CD4^+^ and CD8^+^ T cells and were able to expand CD4^+^CD25^+^Foxp3^+^ T regulatory cells. As the key function of DCs is to promote differentiation of naïve T cells [36], the targeting of DCs by CyaA might represent a strategy of *B. pertussis* towards dampening of the adaptive immune response in the course of infection. Indeed, some subsets of DCs appear to have an important role in protective immunity to respiratory infection with *B. pertussis* [37].

We further report here that CyaA-induced cAMP signaling enhances LPS-mediated dissolution of cell adhesive contacts and promotes chemotactic migration of DCs towards CCL19/21 chemokines. However, we did not observe a correlation between the amount of CCR7 on the DC cell surface after 24 h and the enhanced chemotactic migration of CyaA and LPS-treated DCs, suggesting that the LPS-induced CCR7 expression was sufficient for enhanced migration along the cytokine gradient. Indeed, as documented by the xCelligence measurements, DCs treated with CyaA and LPS exhibited a faster dissolution of adhesive contacts, possibly facilitating migration. It has been shown that actin and myosin inhibitors inhibit the speed of migration but not the directed motion [38] and the integrin-ligand binding properties of cells similarly affects the migration speed [39]. It is therefore possible, that the enhanced motility induced by CyaA in LPS-treated DCs accounts for the higher number of transmigrated DCs in transwell experiments, despite the similar levels of CCR7 expression. It remains to be established, however, to which extent the capacity of CyaA to increase migration might play a role during *B. pertussis* infection *in vivo*, because wild-type *B. pertussis* was shown to inhibit migration of MDDCs towards CCL21 *in vitro* [40]. This was dependent on the presence of pertussis toxin, where the *B. pertussis* strains lacking active pertussis toxins promoted migration of MDDCs [40].

We further demonstrate here that CyaA-treated LPS-stimulated DCs displayed a decreased capacity to stimulate Ag-specific CD4^+^ and CD8^+^ T cells. This finding is consistent with the observations of Boschwitz and co-authors, who demonstrated that CyaA accounts for the suppressive activity of *B. pertussis*-infected monocytes on proliferation of Ag-specific CD4^+^ T cells *in vitro* [41]. Moreover, inhibition of T cell responses has already been demonstrated in the context of *B. pertussis* infection, where T cells from lungs of *B. pertussis*-infected mice were found to be impaired in capacity to respond to *Bordetella* antigens [42]. Recently, it has also been shown that cAMP signaling skews human DC differentiation towards a tolerogenic phenotype and a defective T cell priming capacity [43]. We show here that the impairment of CD4^+^ T cell stimulatory capacity of CyaA-treated DCs was neither due to a decrease in their viability, nor was it due to a decrease in expression of MHC class II and/or co-stimulatory molecules on the cell surface of toxin-treated DCs, or production of inhibitory soluble factors, like IL-10 or prostaglandins. It could, hence, be attributed to a defect in MHC II-restricted presentation of soluble OVA by CyaA-treated DCs, since upon loading with OVA peptide the CyaA-treated DCs enhanced proliferation of OVA-specific CD4^+^ T cells both *in vitro* and *in vivo*. This all excludes a direct inhibitory impact of traces of CyaA in washed DC suspensions on T cell function [44]. Furthermore, we ruled out also the impact of a decreased macropinocytic uptake of OVA by DCs as the major cause of a reduced antigen presentation to T cells [33]. Most of OVA was used here at a low concentration and was thus taken up by the receptor-mediated endocytosis that was insensitive to CyaA/cAMP signaling. Moreover, no reduction in endosomal degradation of OVA in toxin-treated DCs was observed either. Collectively, these results suggest that CyaA-mediated elevation of cellular cAMP concentrations interfered with some steps in the Ag presentation pathway that are downstream to Ag uptake and degradation. The most plausible explanation is that CyaA action caused perturbation of vesicular sorting and trafficking of epitope-loaded MHC II molecules from endosomes to the cell surface. This would go well with our previous observations that exposure to already as little as 10 ng/ml of CyaA does induce massive actin cytoskeleton rearrangements and membrane ruffling in CD11b-expressing myeloid cells [14]. In line with that, cAMP signaling induced by cholera toxin of *V. cholerae* and heat-labile enterotoxin of *E. coli* was also shown to impair presentation of protein or peptide antigens by macrophages or B cell lymphoma, albeit the mechanism has not been analyzed in detail [34], [45], [46].

It has previously been shown that naïve CD8^+^ T cells require a “third signal”, such as IL-12p70, for *in vitro* activation in response to antigenic peptides the [47]. The lack of IL-12p70 signaling may, indeed, explain the observed decrease in naïve CD8^+^ T cell proliferation induced by CyaA-treated BMDCs. However, as IL-12p70 was shown to be dispensable for the activation of memory CD8^+^ T cells [48], it is unlikely that the decrease of IFN-γ-production in human CD8^+^T cells after stimulation with CyaA-treated MDDCs loaded with influenza matrix peptide would be due to a lack of IL-12p70 signaling. CyaA-induced reduction of MHC class I surface expression on BMDCs (Fig. S2) may, however, account for a decreased CD8^+^ T cell stimulatory capacity of CyaA-treated DCs loaded with OVA peptide both *in vitro* and *in vivo*.

Exogenous peptides were shown to take a pinocytic pathway to reach endoplasmic reticulum (ER) [49]. It has further been previously shown that ER stress impairs MHC class I presentation of endogenous as well as of exogenously added peptides by a mouse lymphoma line cells [50]. Moreover, ER-stress induced miR-346 negatively regulates mRNA for the antigen peptide transporter 1 (TAP1), which might explain the reduced MHC class I presentation during ER-stress [51]. It is not known, however, if CyaA or cAMP signaling affects ER functions. Despite of not having observed any impairment of the proteasomal processing function, the present data do not allow to conclude definitively at which level the CyaA activity impaired the capacity of LPS-stimulated DCs to activate CD8^+^ T cells. This issue is currently under investigation and will be subject to a separate study.

*B. pertussis* virulence factors FHA and CyaA were previously shown to induce IL-10-producing T regulatory cells (Tr1) through immunomodulatory effects on DCs [18], [52], [53]. Moreover, it has been shown that CD25^−^Foxp3^+^ T regulatory cells are the predominant suppressive subtype in the lungs of *B. pertussis*-infected mice [54]. In this study we observed that CyaA-treated DCs expanded the numbers of CD4^+^CD25^+^Foxp3^+^ T regulatory cells *in vitro*. However, it remains to be established whether like for the other types of T regulatory cells [3], [52], the CD4^+^CD25^+^Foxp3^+^ T cells participate in subversion of *B. pertussis* clearance and/or limit the immune-mediated pathology during *B.* pertussis infection.

cAMP signaling was recently implicated not only in induction of CD4^+^CD25^+^Foxp3^+^ T cells but also in the development of Th17 cells [55], which together with Th1 cells, are important for clearance of *B. pertussis* from the respiratory tract of infected mice [9]. We found that CyaA-induced cAMP signaling also enhanced the capacity of TLR-activated DCs to stimulate IL-10 and IL-17 production with limited IFN-γ production also by CD8^+^ T cells. IL-17-producing CD8^+^ T cells (Tc17) were, indeed, shown to be involved in modulation of inflammatory immunity during some viral infections [56]. There are, however, no reports yet on the role of Tc17 during *B. pertussis* infection. Although CD8^+^ T cells were found to be dispensable for resolving *B. pertussis* infection in the mouse model [22], [23], in humans IFN-γ producing CD8^+^ T cell responses were detected in a cohort of *B. pertussis*-infected infants and young adults, as well as in vaccinated children [24]. It remains, hence, to be determined whether these cells are induced by the adjuvant activity of *B. pertussis* virulence factors, or whether CD8^+^ T cells actively participate in host defense against *B. pertussis*. CD8^+^ T cells might, indeed, have a role in cytokine production, as well as in cytolytic activity against infected human macrophages, in which *B. pertussis* would survive and grow for some time, as recently documented [57]. Therefore subversion of CD8^+^ T cells responses by CyaA-treated DCs *in vivo* appears to be a relevant hypothesis to test in future studies.

## Supporting Information

Figure S1 **Phenotypic analysis of BMDCs and MDDCs.** (A) BMDC at day 5 (D5) and day 8 (D8) of culture were analyzed for the expression of H2-k^b^, I-A/I-E, CD11c, CD11b, macrophage marker F4/80, granulocyte marker Gr-1 and B cell marker B220 by flow cytometry. DCs at days 7–8 were used for experiments. Histograms are representative of 3 experiments (C57BL/6 mice). The data in graph represent mean values of MFI ± SEM of n = 3. (B) PBMCs at day 0 and MDDCs at day 5 of culture were compared for the expression of CD14, CD1a, CD11c, HLA-DR by flow cytometry. Histograms represent 3 donors showing their variability.
(TIF)

Figure S2 **CyaA differentially modulates TLR-induced maturation and cytokine production of DCs.** BMDCs or MDDCs were left untreated (medium) or incubated with LPS (100 ng/ml BMDCs or 1 µg/ml MDDCs) or in combination with 10 ng/ml CyaA or CyaA-AC^−^. (A) Expression of H-2K^d^, I-A/I-E, CD80, CD86, CD40 and CD54 in living CD11c^+^ BMDCs was determined by flow cytometry after 18 h. Expression of HLA-DR, CD80, CD86, CD40 and CD83 in living CD11c^+^ MDDCs was determined by flow cytometry after 24 h. Values represent the means ± SEM of n = 4–6 or 5 donors, respectively, where the expression of molecules by LPS-stimulated DCs (LPS) was set as 1.0 (* *p*<0.05). (B) Secretion of IL-10 and IL-12p70 was determined from BMDC culture supernatants by ELISA after 18 h and from MDDC culture supernatants by Luminex after 24 h. Values represent the means ± SEM of n = 4 or 5 donors, respectively.
(TIF)

Figure S3 **OVA protein at low concentration is taken up by TLR-stimulated DCs solely via receptor-mediated endocytosis which is unaffected by CyaA.** BMDCs left untreated, incubated with LPS (100 ng/ml) alone or in combination with 10 ng/ml of CyaA for 30 min, followed by the incubation with mannan (1 mg/ml) or Poly(I) (10 µM) to block receptor-mediated endocytosis for 30 min. After that OVA-FITC (5 µg/ml) was added to samples for 30 min. The antigen uptake in living CD11c^+^ cells was determined by flow cytometry. Values represent means ± SEM of n = 4 where OVA-FITC taken up by LPS-treated DC (medium) was set to 100% of MFI.
(TIF)

Figure S4 **Analysis of CD4^+^CD25^+^Foxp3^+^ T regulatory cells.** Gating strategy for mouse (A) and human (B) T regulatory cells is shown.
(TIF)

Figure S5 **BMDCs treated with CyaA and LPS induce IL-10 and IL-17-secreting CD4^+^ T cells with limited IFN-γ production.** BMDCs were left untreated, incubated with LPS (100 ng/ml) alone or in combination with CyaA or CyaA-AC^−^ at 10 ng/ml in the presence of OVA at 2.5 µg/ml for 4 h prior to co-cultivation with naïve CFSE-labeled OT-II CD4^+^ T cells. After 72 h IL-10, IL-17 and IFN-γ production in DC-CD4^+^ T cell culture supernatants was determined by ELISA. Values represent means ± SEM of n = 3 (* *p*<0.05).
(TIF)

Figure S6 **CyaA induces phenotypic maturation of DCs.** (A) MDDCs and BMDCs (2×10^5^/sample) were left untreated, incubated with LPS alone or with CyaA or CyaA-AC^−^ at 10 ng/ml for 24 h at 37°C. The expression of H2-K^b^, I-A/I-E or HLA-DR, CD80, CD83, CD40 and CD54 was detected by flow cytometry. The data represent mean values ± SEM of n = 4 or 5 donors (* *p*<0.05). (B) The cytokines in supernatants of cell culture were detected by Luminex (MDDCs) or by antibody array (RayBio; BMDCs). The data represent mean values ± SEM of n = 4 or 5 donors (* *p*<0.05). (C) Mixed lymphocyte reaction: MDDCs were left untreated, incubated with LPS alone or with CyaA or CyaA-AC^−^ at 10 ng/ml for 24 h at 37°C. Allogeneic CFSE-labeled T lymfocytes were added at T cell: MDDC ratio of 10 : 1. IL-2 (50 U/ml) was added on day 3. On day 4, the proliferation of T cells was determined by flow cytometry. BMDCs were incubated with CyaA or CyaA-AC^−^ at 10 ng/ml for 24 h at 37°C. The proliferation of allogeneic (BALB/c mice) CFSE-labeled T cells was determined by flow cytometry after 72 h. The histograms are representative and the graphs show mean values ± SEM of n = 4 or 5 donors (* *p*<0.05).
(TIF)
