# Supplementary figures and images for: Bordetella Adenylate Cyclase Toxin Differentially Modulates Toll-Like Receptor-Stimulated Activation, Migration and T Cell Stimulatory Capacity of Dendritic Cells
Source: PLoS One. 2014 Aug 1;9(8):e104064. doi: 10.1371/journal.pone.0104064 (PMC4118975; doi:10.1371/journal.pone.0104064)

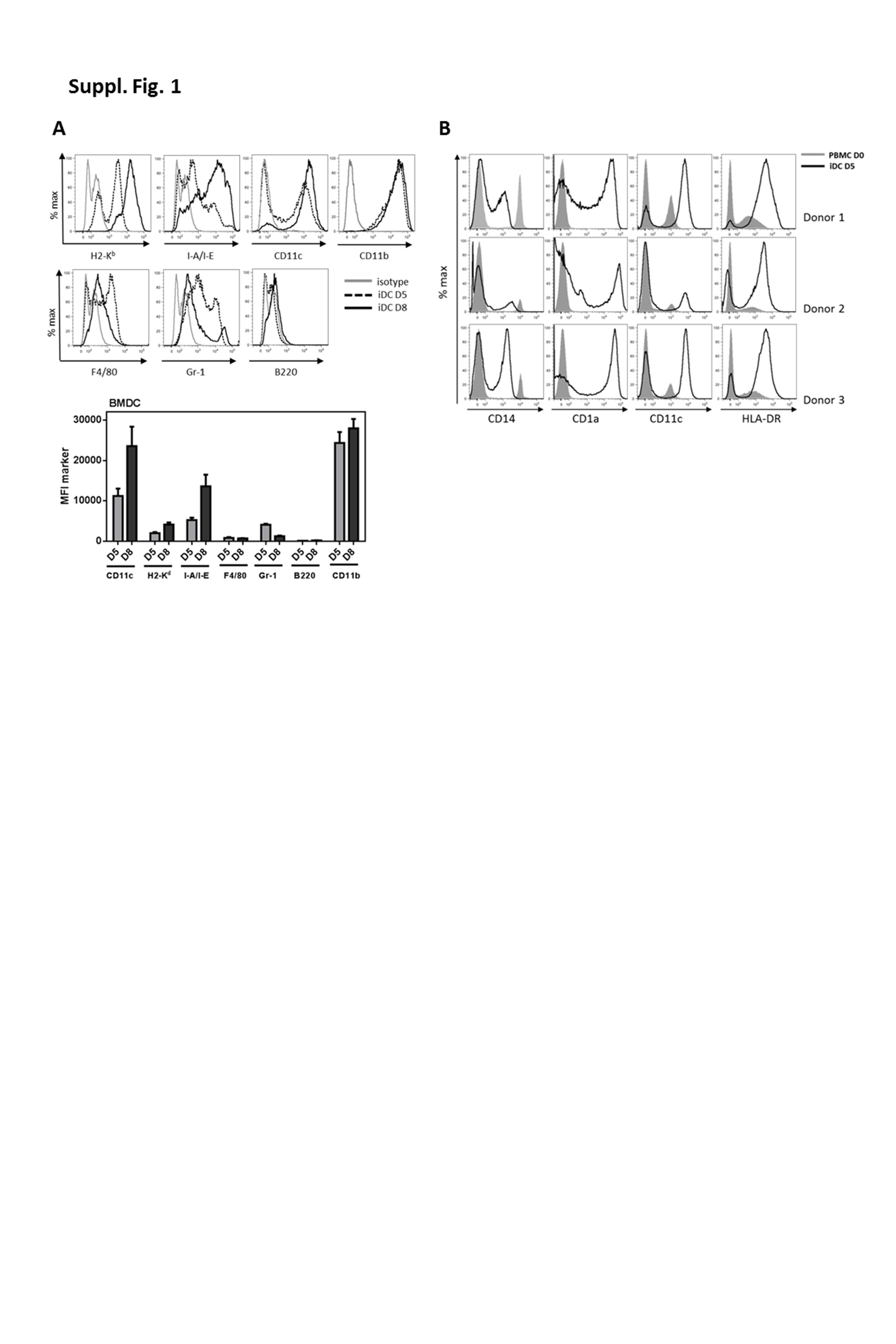

Supplement: Figure S1 — Phenotypic analysis of BMDCs and MDDCs. (A) BMDC at day 5 (D5) and day 8 (D8) of culture were analyzed for the expression of H2-kb, I-A/I-E, CD11c, CD11b, macrophage marker F4/80, granulocyte marker Gr-1 and B cell marker B220 by flow cytometry. DCs at days 7–8 were used for experiments. Histograms are representative of 3 experiments (C57BL/6 mice). The data in graph represent mean values of MFI ± SEM of n = 3. (B) PBMCs at day 0 and MDDCs at day 5 of culture were compared for the expression of CD14, CD1a, CD11c, HLA-DR by flow cytometry. Histograms represent 3 donors showing their variability. (TIF) [file pone.0104064.s001.tif]

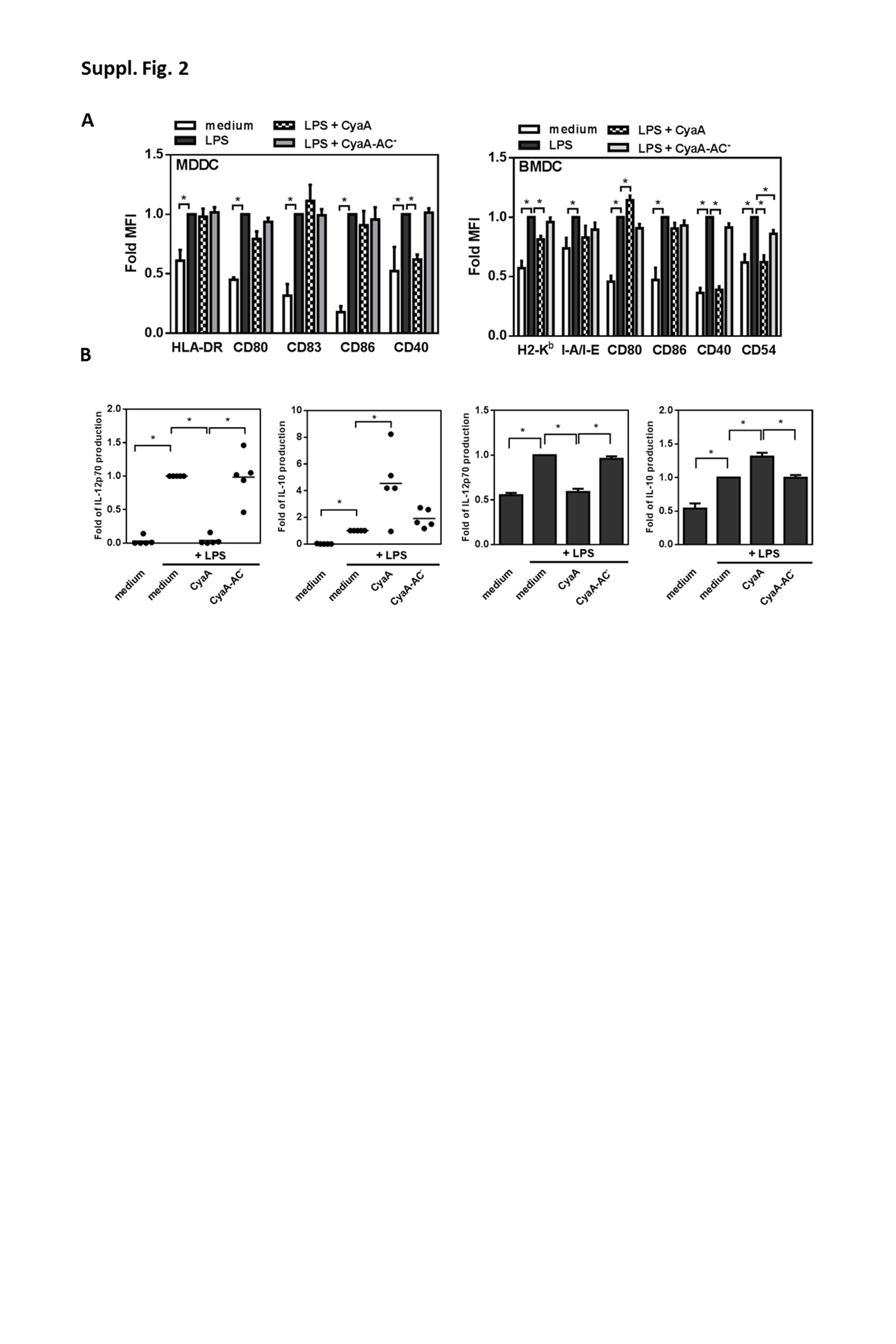

Supplement: Figure S2 — CyaA differentially modulates TLR-induced maturation and cytokine production of DCs. BMDCs or MDDCs were left untreated (medium) or incubated with LPS (100 ng/ml BMDCs or 1 µg/ml MDDCs) or in combination with 10 ng/ml CyaA or CyaA-AC−. (A) Expression of H-2Kd, I-A/I-E, CD80, CD86, CD40 and CD54 in living CD11c+ BMDCs was determined by flow cytometry after 18 h. Expression of HLA-DR, CD80, CD86, CD40 and CD83 in living CD11c+ MDDCs was determined by flow cytometry after 24 h. Values represent the means ± SEM of n = 4–6 or 5 donors, respectively, where the expression of molecules by LPS-stimulated DCs (LPS) was set as 1.0 (* p<0.05). (B) Secretion of IL-10 and IL-12p70 was determined from BMDC culture supernatants by ELISA after 18 h and from MDDC culture supernatants by Luminex after 24 h. Values represent the means ± SEM of n = 4 or 5 donors, respectively. (TIF) [file pone.0104064.s002.tif]

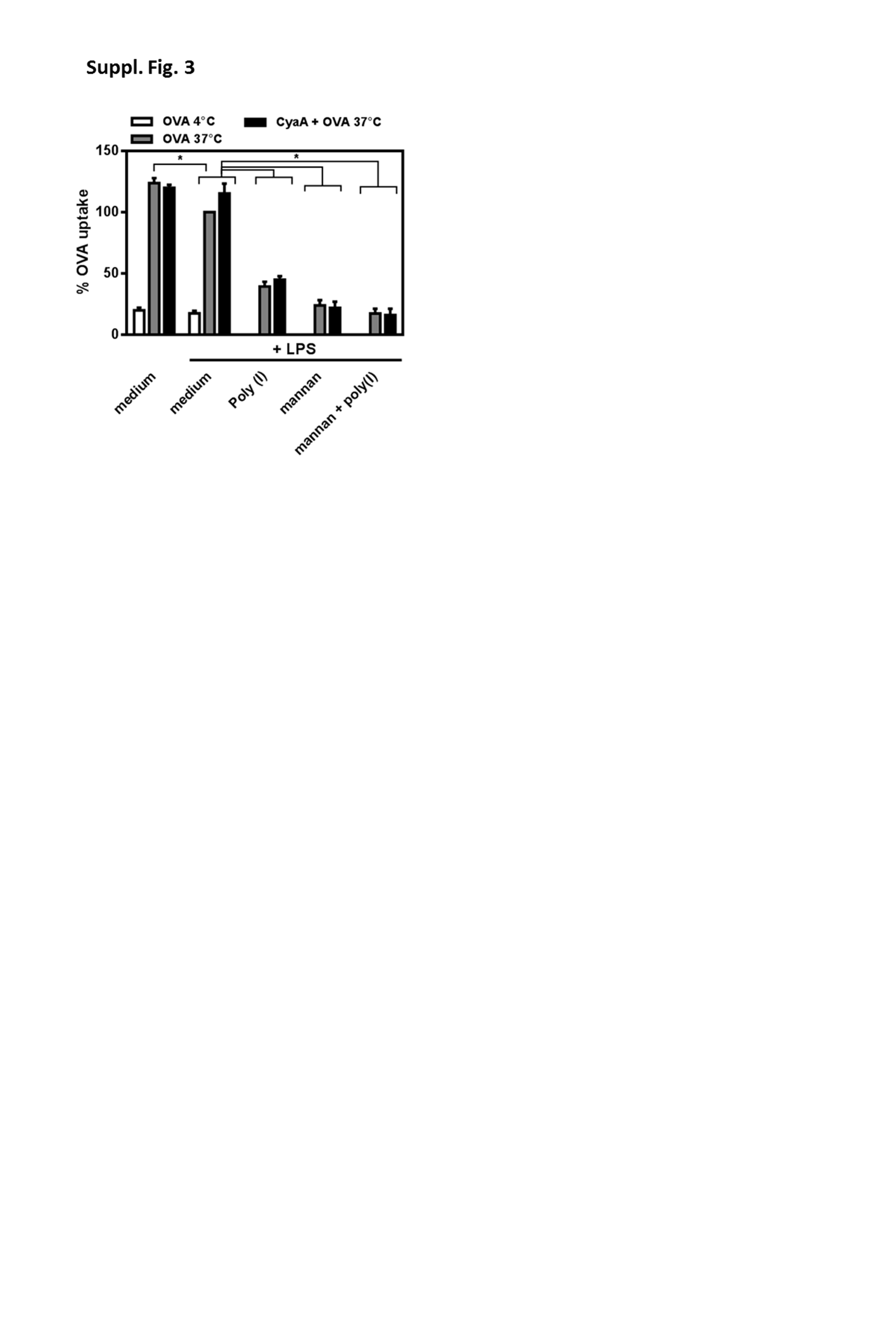

Supplement: Figure S3 — OVA protein at low concentration is taken up by TLR-stimulated DCs solely via receptor-mediated endocytosis which is unaffected by CyaA. BMDCs left untreated, incubated with LPS (100 ng/ml) alone or in combination with 10 ng/ml of CyaA for 30 min, followed by the incubation with mannan (1 mg/ml) or Poly(I) (10 µM) to block receptor-mediated endocytosis for 30 min. After that OVA-FITC (5 µg/ml) was added to samples for 30 min. The antigen uptake in living CD11c+ cells was determined by flow cytometry. Values represent means ± SEM of n = 4 where OVA-FITC taken up by LPS-treated DC (medium) was set to 100% of MFI. (TIF) [file pone.0104064.s003.tif]

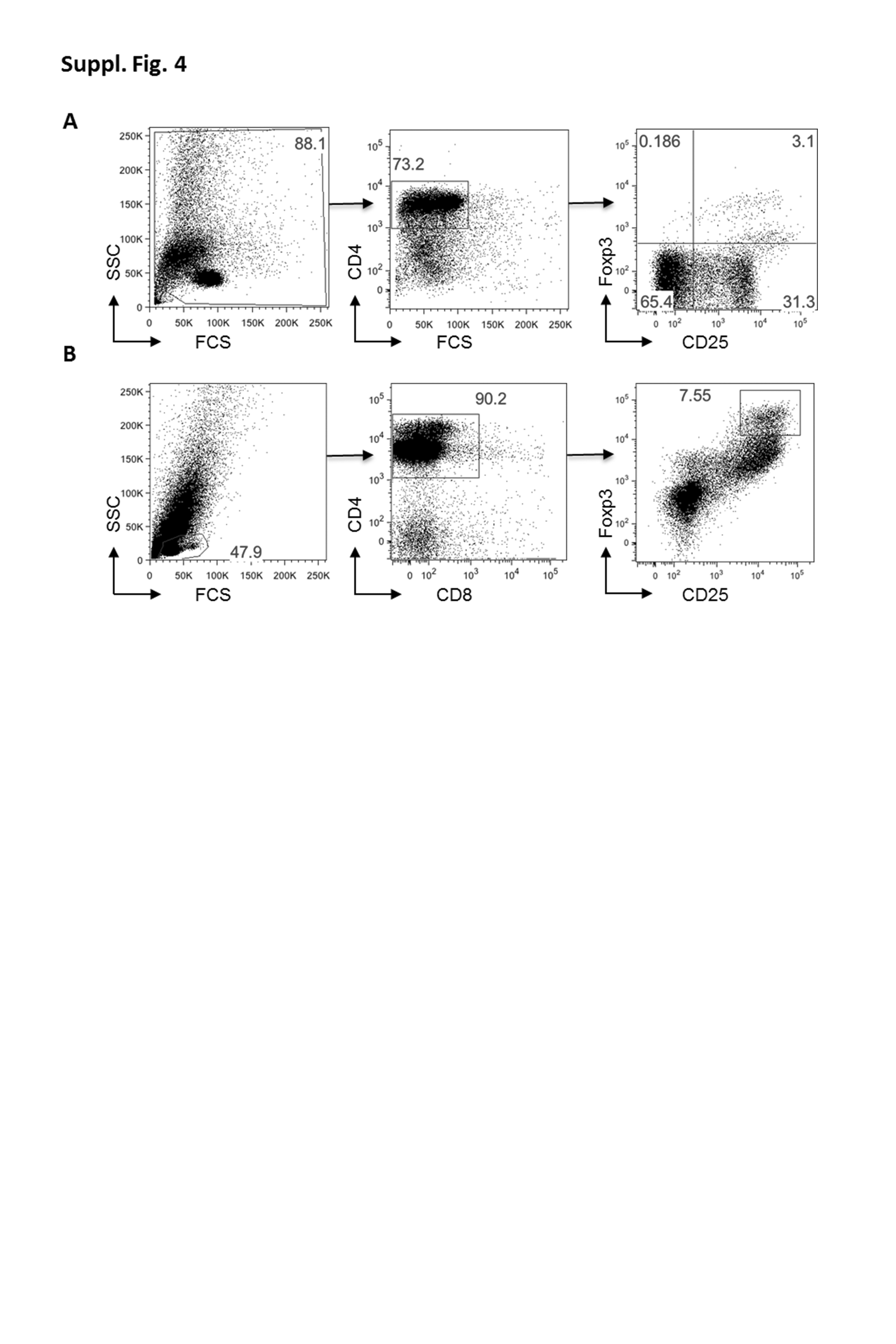

Supplement: Figure S4 — Analysis of CD4+CD25+Foxp3+ T regulatory cells. Gating strategy for mouse (A) and human (B) T regulatory cells is shown. (TIF) [file pone.0104064.s004.tif]

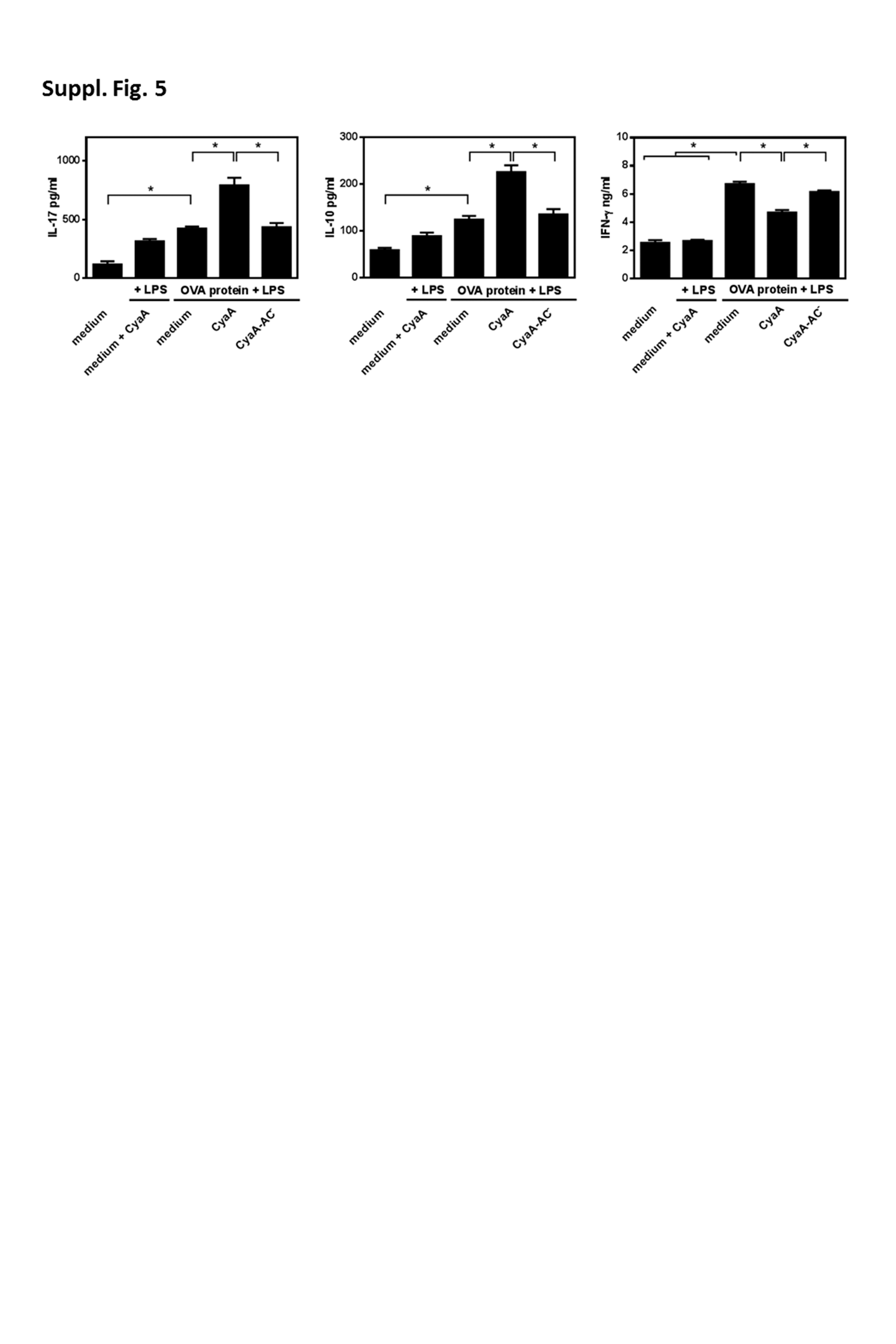

Supplement: Figure S5 — BMDCs treated with CyaA and LPS induce IL-10 and IL-17-secreting CD4+ T cells with limited IFN-γ production. BMDCs were left untreated, incubated with LPS (100 ng/ml) alone or in combination with CyaA or CyaA-AC− at 10 ng/ml in the presence of OVA at 2.5 µg/ml for 4 h prior to co-cultivation with naïve CFSE-labeled OT-II CD4+ T cells. After 72 h IL-10, IL-17 and IFN-γ production in DC-CD4+ T cell culture supernatants was determined by ELISA. Values represent means ± SEM of n = 3 (* p<0.05). (TIF) [file pone.0104064.s005.tif]

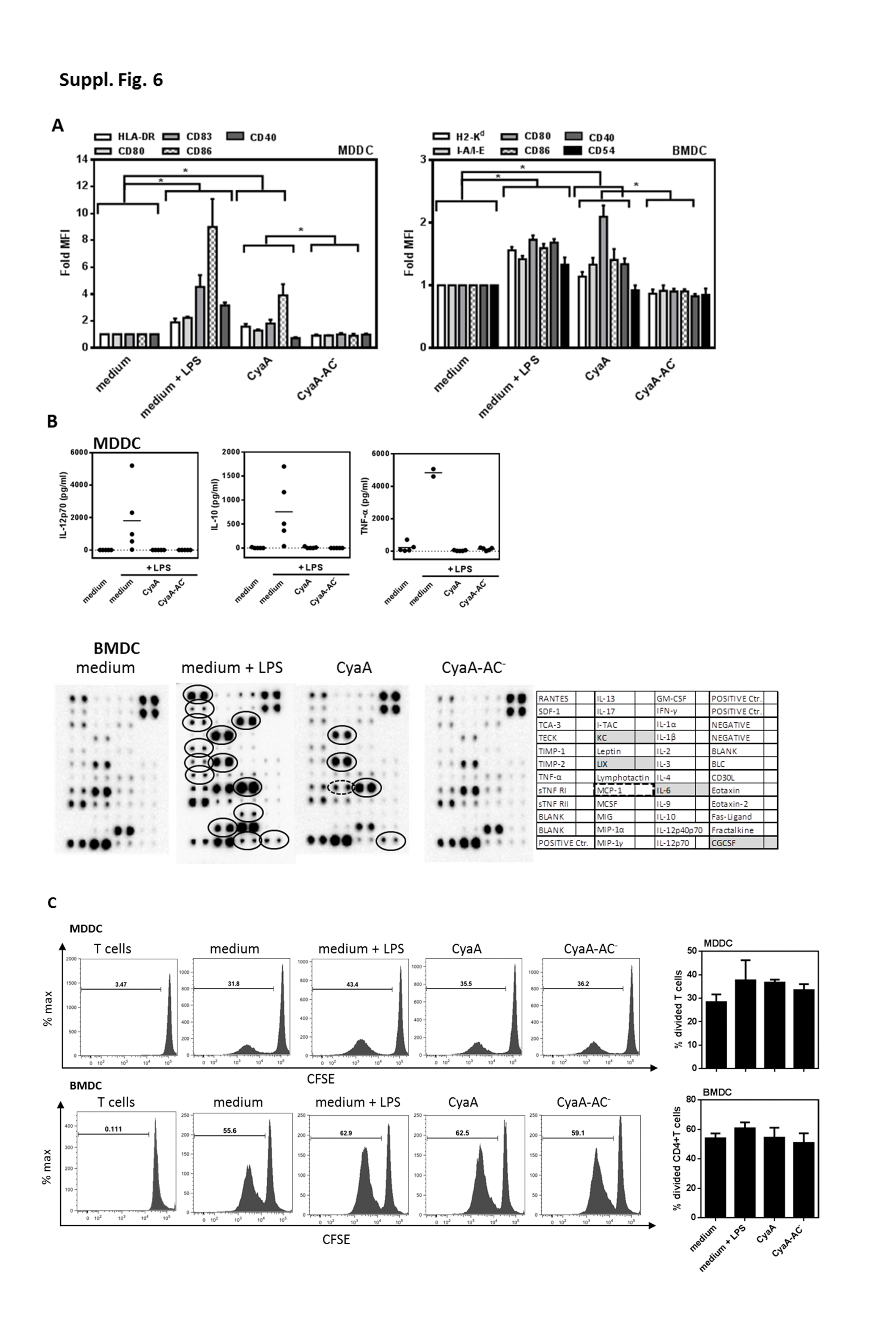

Supplement: Figure S6 — CyaA induces phenotypic maturation of DCs. (A) MDDCs and BMDCs (2×10^5^/sample) were left untreated, incubated with LPS alone or with CyaA or CyaA-AC− at 10 ng/ml for 24 h at 37°C. The expression of H2-Kb, I-A/I-E or HLA-DR, CD80, CD83, CD40 and CD54 was detected by flow cytometry. The data represent mean values ± SEM of n = 4 or 5 donors (* p<0.05). (B) The cytokines in supernatants of cell culture were detected by Luminex (MDDCs) or by antibody array (RayBio; BMDCs). The data represent mean values ± SEM of n = 4 or 5 donors (* p<0.05). (C) Mixed lymphocyte reaction: MDDCs were left untreated, incubated with LPS alone or with CyaA or CyaA-AC− at 10 ng/ml for 24 h at 37°C. Allogeneic CFSE-labeled T lymfocytes were added at T cell: MDDC ratio of 10 : 1. IL-2 (50 U/ml) was added on day 3. On day 4, the proliferation of T cells was determined by flow cytometry. BMDCs were incubated with CyaA or CyaA-AC− at 10 ng/ml for 24 h at 37°C. The proliferation of allogeneic (BALB/c mice) CFSE-labeled T cells was determined by flow cytometry after 72 h. The histograms are representative and the graphs show mean values ± SEM of n = 4 or 5 donors (* p<0.05). (TIF) [file pone.0104064.s006.tif]
